# Non-Random Enrichment of Single-Nucleotide Polymorphisms Associated with Clopidogrel Resistance within Risk Loci Linked to the Severity of Underlying Cardiovascular Diseases: The Role of Admixture

**DOI:** 10.3390/genes14091813

**Published:** 2023-09-17

**Authors:** Mariangeli Monero-Paredes, Roberto Feliu-Maldonado, Kelvin Carrasquillo-Carrion, Pablo Gonzalez, Igor B. Rogozin, Abiel Roche-Lima, Jorge Duconge

**Affiliations:** 1 Department of Pharmacology and Toxicology, School of Medicine, University of Puerto Rico, Medical Sciences Campus, San Juan 00936, Puerto Rico; mariangeli.monero@upr.edu (M.M.-P.); pablo.gonzalez3@upr.edu (P.G.); 2 Research Centers in Minority Institutions Program, Center for Collaborative Research in Health Disparities, Academic Affairs Deanship, University of Puerto Rico, Medical Sciences Campus, San Juan 00936, Puerto Rico; roberto.feliumaldonado@upr.edu (R.F.-M.); kelvin.carrasquillo@upr.edu (K.C.-C.); abiel.roche@upr.edu (A.R.-L.); 3 Computational Biology Branch, National Center for Biotechnology Information (NCBI), National Library of Medicine (NLM), National Institutes of Health (NIH), Rockville Pike MSC 3830, Bethesda, MD 20894, USA; ibrogozin@gmail.com; 4 Department of Pharmaceutical Sciences, School of Pharmacy, University of Puerto Rico, Medical Sciences Campus, San Juan 00936, Puerto Rico

**Keywords:** clopidogrel, Caribbean Hispanics, biomarkers, cardiovascular diseases, pharmacogenomics, ancestry

## Abstract

Cardiovascular disease (CVD) is one of the leading causes of death in Puerto Rico, where clopidogrel is commonly prescribed to prevent ischemic events. Genetic contributors to both a poor clopidogrel response and the severity of CVD have been identified mainly in Europeans. However, the non-random enrichment of single-nucleotide polymorphisms (SNPs) associated with clopidogrel resistance within risk loci linked to underlying CVDs, and the role of admixture, have yet to be tested. This study aimed to assess the possible interaction between genetic biomarkers linked to CVDs and those associated with clopidogrel resistance among admixed Caribbean Hispanics. We identified 50 SNPs significantly associated with CVDs in previous genome-wide association studies (GWASs). These SNPs were combined with another ten SNPs related to clopidogrel resistance in Caribbean Hispanics. We developed Python scripts to determine whether SNPs related to CVDs are in close proximity to those associated with the clopidogrel response. The average and individual local ancestry (LAI) within each locus were inferred, and 60 random SNPs with their corresponding LAIs were generated for enrichment estimation purposes. Our results showed no CVD-linked SNPs in close proximity to those associated with the clopidogrel response among Caribbean Hispanics. Consequently, no genetic loci with a dual predictive role for the risk of CVD severity and clopidogrel resistance were found in this population. Native American ancestry was the most enriched within the risk loci linked to CVDs in this population. The non-random enrichment of disease susceptibility loci with drug-response SNPs is a new frontier in Precision Medicine that needs further attention.

## 1. Introduction

Caribbean Hispanics are underrepresented in genetic studies, limiting knowledge of genetic risk factors for both CVD severity and a poor response to clopidogrel [1]. Most of the characterized SNPs associated with CVDs, such as acute coronary syndrome (ACS), coronary artery disease (CAD), and peripheral artery disease (PAD), have been identified in patients of predominantly European ancestry [2,3]. Cardiovascular risk factors to inform the disease severity (e.g., type-2 diabetes, a left ventricular ejection fraction (LVEF) < 30%, an adjoined length of stent(s) ≥ 30 mm, smoking) are also predictive biomarkers of a poor response to clopidogrel [4,5,6,7]. However, there is no evidence of significant associations between these markers at the genomic level. We hypothesized that genetic loci linked to these risk factors and the response to clopidogrel are inherited together or associated with each other.

Unlike Mendelian-type monogenic conditions with large effect sizes, complex traits affected by many genes with moderate-to-small effect sizes each (e.g., CVDs) are less likely to transfer across populations. Given their diverse evolutionary histories, resulting in differential allele frequency distributions and varying effect sizes across ethnicities of distinct ancestry and degree of admixture, the role of unique genomic architectures on transferability is critical and must be accounted for. Indeed, the poor transferability of findings from European-centered genomic associations to Hispanics influences how well true causal variants are captured by SNPs identified in Europeans and ultimately exacerbate health inequities. To mitigate the impact of population-specific attributes and the resulting poor transferability of genomic results on health equity, it is imperative to take ancestry into account and expand current studies to admixed populations in which the traits of interest are highly prevalent. Caribbean Hispanics are an admixed population with high genetic heterogeneity that gives rise to phenotypic diversity. By accounting for the local ancestry (LA) effect, we may also uncover novel variants associated with the clopidogrel response. Therefore, inferring genetic ancestry while assessing the relationship between ACS/CAD- and PAD-associated SNPs and those associated with clopidogrel resistance is important for reducing health disparities in this underrepresented population.

Bioinformatic computational tools have been developed to facilitate the study of the global ancestry composition in individuals. This has permitted us to understand the evolutionary and migration patterns of populations [8,9]. Because the SNP frequency can change from one population to another, it could serve as a genetic signature or landmark in population genetic studies. In addition, advancement in local-ancestry inference (LAI) tools has continued, helping elucidate the disease risk and optimal drug therapy in populations [10,11]. LAI is described as the ancestry in a particular location of the chromosome of an individual. LAI works by matching a segment of the reference population to a segment of the chromosome of an admixed individual and assigning the respective ancestry. In the case of Caribbean Hispanics, the reference populations include European, African, and Native American.

LAI results will also depend on the applied method. Various methods for LAI have been developed, including the Local Ancestry in adMixed Populations (LAMP-LD) [12], RFMix [13], Efficient Local Ancestry Inference (ELAI) [14], and MOSAIC [15] software. LAMP-LD is a software package for the inference of the locus-specific ancestry in recently admixed populations [12]. RFMix is a powerful discriminative modeling approach for rapid and robust local-ancestry inference [13]. ELAI performs local-ancestry inference for admixed individuals [14], whereas MOSAIC infers segments of ancestry and characterizes admixture events involving an arbitrary number of genetically distinct groups put together [15]. However, a study showed that the most accurate method is RFMix [16]. Also, a study comparing LAMP-LD and RFMix showed that RFMix had a shorter run time [17].

Studies inferring local ancestry by using RFMix have been carried out on Hispanics, but not on a cohort of Caribbean Hispanic patients with ACS, CAD, and PAD receiving clopidogrel treatment [18,19]. In this study, we ascertained ancestry enrichments at relevant CV loci, as well as non-random associations between clopidogrel-related genomic markers and those linked to the severity of CV diseases, in admixed Caribbean Hispanics.

## 2. Materials and Methods

### 2.1. Study Population

Caribbean Hispanic patients, self-identified as Cuban, Dominican, or Puerto Rican, on treatment with clopidogrel (75 mg/day), were included in the study. The participants were recruited at five medical facilities across the Commonwealth of Puerto Rico: The Cardiovascular Center of Puerto Rico and the Caribbean, San Francisco Hospital, Dr. Hilton Franqui Clinics, Pavia Hospital, and UPR Hospital Dr. Federico Trilla. All participants met the inclusion/exclusion criteria (Table 1) and signed an informed consent approved by the Institutional Review Board (IRB protocol number A4070417). Relevant demographic and clinical data were collected along with a blood sample in two 3.0 mL 3.2% sodium citrate tubes. Medication adherence was assessed via self-report and record reviews, using the highly validated and reliable four-item Morisky Medication Adherence Scale (MMAS-4), as described elsewhere [20]. A flow diagram of the study design is depicted in Figure S1.

### 2.2. Identification of Variants

A literature assessment was performed to identify 50 SNPs that were related to CVDs, such as ACS, CAD, and PAD. No date restrictions were employed. We used the NHGRI-EBI GWAS Catalog [22] (https://www.ebi.ac.uk/gwas/, accessed on 29 April 2021) as the search engine for SNPs associated with each of the three CVDs. First, we searched using the key term “acute coronary syndrome” and we arranged the SNPs in order of *p*-value significance, followed by the selection of the 20 most significant SNPs. Then, we repeated the same procedure using the key term “coronary artery disease”. Some SNPs for CAD were already included for ACS; therefore, we evaluated and selected the SNP with the highest *p*-value for the corresponding condition. For PAD, we used the key term “peripheral artery disease” and we selected only the first 10 most significant SNPs, as the minority of the patients were on treatment with clopidogrel for PAD. The SNP positions were acquired as the IDs for further analysis steps. The positions were based on GRCh37 as the reference genome build. In addition, 10 SNPs corresponding to the top 10 risk locus signals from a GWAS on high on-treatment platelet reactivity (HTPR) performed on Caribbean Hispanic patients on clopidogrel were included (unpublished data). We evaluated whether any of these SNPs associated with either CVDs or clopidogrel resistance could also be expression quantitative trait loci (eQTLs). To this purpose, we employed the Gtex Portal (https://gtexportal.org/home/, accessed on 29 April 2021), an online tool used to evaluate possible eQTLs from our variants of interest.

### 2.3. Local-Ancestry Inference

Genotyped data were used to estimate haplotypes using Shapeit v2.17 software. RFMix software v1 was performed to infer local ancestry by using the code available at https://github.com/indraniel/rfmix/blob/master/RunRFMix.py (accessed on 7 October 2021). For the reference population, we used genotyped data from 107 individuals of the Iberian population in Spain (IBS) to represent European ancestry, 61 individuals of Yoruba ancestry in Ibadan, Nigeria, (YRI) to represent African ancestry, and 103 Continental Native American individuals from the America population of the Human Genome Diversity Project (HGDP) to represent Native American (NA) ancestry. The IBS and YRI populations came from the NHGRI Sample Repository for Human Genetic Research of the Coriell Institute (https://www.internationalgenome.org/data-portal/population, accessed on 7 October 2021). The America population was defined as Native American, and the samples came from Surui and Karitiana in Brazil, Piapoco and Colombian in Colombia, and Maya and Pima in Mexico (https://www.internationalgenome.org/data-portal/data-collection/hgdp, accessed on 7 October 2021).

### 2.4. Python Scripts

Because computational tools have allowed us to analyze high-throughput data, we assessed the non-random enrichment in the association between clopidogrel resistance and the severity of underlying ACS, CAD, and PAD by determining the close proximity (±0.5 cM) between SNPs. Three Python scripts were developed fittingly for the purpose of each objective: (1) to estimate the local ancestral proportions by variant, (2) to determine whether variants of interest are found in close proximity (±0.5 cM), and (3) to acquire random variants from an existing dataset. The scripts can be found at https://github.com/mariangelimonero/Non-random_enrichment (accessed on 13 June 2022). The open-source Python v3.10.2 application was used for this purpose.

#### 2.4.1. Ancestry Search

Ancestry data from each individual were obtained via RFMix software (https://github.com/indraniel/rfmix, accessed on 13 June 2022) [17]. The Python script was developed to determine the ancestry within each locus genotyped or imputed across the whole genome of the 510 samples and available in the dataset. As the input file, we used the information about the location files in cM and the forward–backward files containing ancestry information for each variant. The script consisted of opening the location files and forward–backward files of each chromosome and appending them to a list. This information was used to gather ancestry data for the variants and was stored as an “all_chr.anc” file extension for each of the samples. A text file with the samples to be included in the analysis was set on the script. Ancestries were matched with the given sample names by appending them to a list. The rs number and the chromosome number were obtained and appended to their respective lists. The two lists were then transformed into a data frame and then concatenated into a single data frame. Also, the list containing the information about the position and cM was transformed into a data frame and concatenated with the data frame of the rs number and chromosome, creating a single data frame. A column with the calculation of the average ancestry by variant was created. Finally, the previously created data frames were concatenated, creating a file with the columns of rs numbers, chromosomes, positions, cM values, average ancestries, and subsequent samples.

#### 2.4.2. Random Search

A Python script was developed to randomly find and select another 60 variants from the dataset. A text file was provided as input, with the positions of the rs numbers of interest that we did not want to include as random variants. The ancestry file developed in the previous ancestry script was used to provide information about ancestry from the 60 random signals. The script was developed to search for n random signals. It was designed to append all rs numbers in a list, excluding the ones of interest. The function shuffle was applied to the list. The output was given with the quantity of variants desired and the columns of rs numbers, chromosomes, positions, cM values, average ancestries, and subsequent samples.

#### 2.4.3. Bracket Search

A Python script was developed to find two variants of interest in proximity (i.e., located within ± 0.5 cM of each other). A list of the variants of interest and the ancestry file developed in the previous ancestry search step were used as the input file. The variants of interest and the ancestry file were added to individual lists. The intersection function was used to determine the variants of interest that were available in the ancestry list. If the intersection was true, then the matching variants were written in the output file. The output file was then converted into a data frame, and we determined whether the variants were +0.5 or −0.5 cM far apart from each other. Repeated matching pair variants were eliminated. This created a csv file with only the position and cM of the variants in proximity. The data from ancestry and matching variants were converted into a csv file with columns of rs numbers, chromosomes, positions, cM values, and subsequent samples.

### 2.5. Statistical Analysis

To characterize the study cohort, a descriptive analysis of the demographics and clinical parameters was performed. Categorical data were summarized as frequencies and percentages. Continuous variables were reported as mean ± standard deviation (SD) and standard error of mean (SEM), minimum and maximum values, and median. Statistically significant differences in the non-random enrichments between groups were assessed, using either chi-square or Fisher’s exact probability tests for categorical variables. All statistical analyses were performed using PLINK v1.9.

## 3. Results

### 3.1. Data Cohort

This is a secondary analysis of a dataset from a multicenter clinical study protocol (NCT03419325) [20]. For this purpose, a subcohort of 510 Caribbean Hispanic patients on treatment with clopidogrel and with full genetic and clinical data available was used. Patients were diagnosed with CVDs such as ACS, CAD, and/or PAD. All diagnoses were obtained from the patient’s electronic medical records at each participating medical facility using the corresponding ICD-9/10-CM codes, which were subsequently confirmed by the study cardiologists. Patients receiving either a 600 mg or 300 mg loading dose of clopidogrel followed by a maintenance dose of 75 mg daily or who started with a 75 mg daily maintenance dose (i.e., alone or as a component of dual antiplatelet therapy (DAPT) in mainly post-PCI patients with ACS or stable CAD with elective stenting) were enrolled in the study between January 2018 and June 2020 from five different medical facilities across the island. Enrollment criteria (Table 1) were minimal, with the goal of gathering an “all comers” population representative of real-world clinical practice. We observed high adherence scores (>85%) during the study period. Participants with a significant lack of adherence to therapy were removed from subsequent analyses. The cohort was genotyped for approximately 1.4 million SNPs with the Infinium^TM^ Multi-Ethnic AMR/AFR Genotyping Array (MEGA) BeadChip by Illumina (San Diego, CA, USA).

Table 2 describes the baseline characteristics of the participants (*n* = 510). The average age of all the participants in this study was 68 years old, with 55% identified as male. Overall, 78.3% were aged 60 years old or above and 18.6% were within the 45–59-year-old range. Largely, most were middle-aged males, with a high prevalence of conventional risk factors (i.e., overweight (28.4 kg/m^2^), hypertension (83.9%), hypercholesterolemia/dyslipidemias (71.9%), and type-2 diabetes (54.8%), among others). Furthermore, 20% of participants were on proton pump inhibitors (PPIs) (mainly pantoprazole), whereas statins and calcium channel blockers (CCBs) were prescribed in 79.2% and 26.8% of patients, respectively. Patients who were taking aspirin administered as part of DAPT represented 63.3% of the total cohort. In 75.9% of participants, clopidogrel was given for stable CAD/ACS indication. No differences in the baseline characteristics (e.g., comorbidities) existed among the patients after stratifying by carrier status or indication.

### 3.2. Expression Quantitative Trait Locus (eQTL) SNPs

The intronic variant rs55791371 (Table 3 and Table 4, and Table S9) is an eQTL that resides within the gene encoding the BRG1 protein. The rs9349379 SNP is another identified eQTL variant located at an intronic position in the phosphatase and actin regulator 1 (PHACTR1) gene (Table 4 and Table S3). Moreover, the rs10455872, rs118039278, and rs140570886 SNPs are also eQTL variants associated with thrombus formation (Table 3 and Table 4, and Table S3). In addition, the rs55791371 variant (Table 3 and Table 4, and Table S9) is an eQTL for the SLC44A2 gene that encodes a choline transporter protein.

### 3.3. Non-Random Enrichment of SNPs

After a literature review, we identified 20 SNPs associated with ACS, 20 SNPs associated with CAD, and 10 SNPs associated with PAD (Table 3). Although some SNPs were repeated in two different CVDs, we kept the SNP for the condition with the strongest evidence. Additionally, we obtained the ten top signals from a GWAS on clopidogrel resistance among Caribbean Hispanic patients in our laboratory (Table 3). The total of these 60 SNPs was analyzed with the Python script developed for the bracket search.

To determine whether the SNPs associated with a poor clopidogrel response in our cohort (i.e., top 10 GWAS hits) are occurring close to or within the same genetic loci of SNPs linked to CVDs, our Python script was used. Not a single SNP linked to the severity of CVDs was found either upstream (+0.5 cM) or downstream (−0.5 cM) of the corresponding loci of any SNP associated with clopidogrel resistance. Additional functional information of these 60 SNPs was assessed and is reported in Tables S1–S11 (Supplementary Materials).

### 3.4. Local-Ancestry Inferences (LAIs)

**Figure 1:**
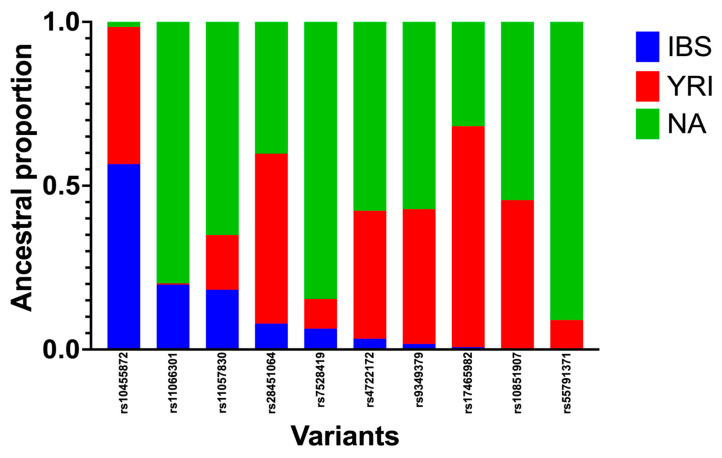
Ancestry proportions around the 10 SNPs associated with CVDs (ACS, CAD, and PAD) using the Python script for ancestry search. Genotyping data of 510 Caribbean Hispanics were obtained via Infinium Multi-Ethnic AMR/AFR Genotyping Array (MEGA) BeadChip by Illumina^®^. RFMix software was used to estimate locus-specific ancestries by using reference populations from Iberians, Yoruba, and Native Americans. A Python script was developed to infer ancestry proportions from the list of SNPs of interest.

**Figure 2:**
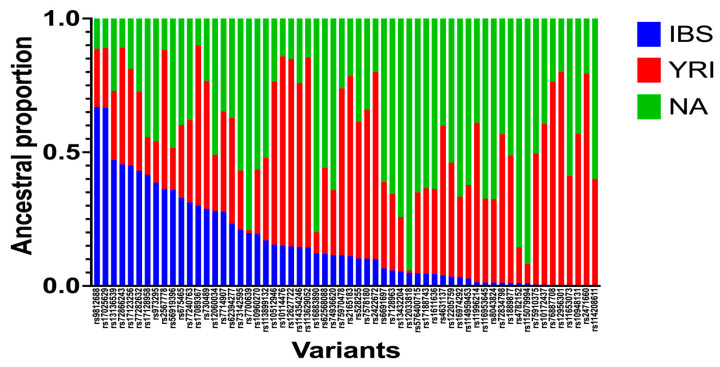
Ancestral proportions of 60 SNPs generated from ancestry search Python script. A total of 510 Caribbean Hispanic patients were genotyped via the Illumina MEGA chip microarray. Genotyped data were phased using Shapeit software. Local-ancestry inferences were obtained via RFMix software. A Python script was designed to identify ancestral proportions for available variants. Ancestries are represented as Iberian population (IBS), Yoruba population (YRI), and Native population from America (NA).

The developed script for the ancestry search was able to identify the locus-specific enrichment of ancestries related to the European (Iberians), African (Yoruba), and Native American contributions within each of the selected SNPs in each individual. However, we were only able to infer the ancestry for 10 out of the 50 SNPs associated with CVDs (Table 4; Figure 1). This is because the available databases from the reference parental populations lacked the necessary information on these other 40 SNPs genotyped in our cohort of Caribbean Hispanics, which is required by RFMix software to make LAIs. As a control group, we also estimated the ancestry proportions at 60 random SNPs for the purpose of ancestry enrichment comparison. Our results showed ≥50% enrichment of Native American, African, and European ancestries in 25, 20, and 2 of these 60 variants, respectively (Table 5; Figure 2).

## 4. Discussion

Understanding the genetic architecture of an admixed population is key to the prevention, diagnosis, and treatment of CVDs. Previous GWASs have identified SNPs associated with the risk of CVDs [24,45,46]. Although these studies are informative enough about potential risk markers, admixed populations are neglected because most of the participants are of European descent. The top ten signals with the lowest *p*-values in our GWAS on the HTPR in CV patients on clopidogrel were used for testing their non-random enrichment with risk variants of CVDs.

SNP Annotation and Tissue Expression: Seven of these signals (Table 3) are located close to a cluster of cytochrome P450 genes on chromosome 10q23.33, which is where the highly polymorphic *CYP2C19* gene is mapped to (reference GRCh38.p14 assembly: from 94,762,681 to 94,855,547). These seven SNPs form a cluster ~8 kb long on chromosome 10, suggesting they are segregated. The closest gene is located more than 1 Mbp away from this cluster, implying possible distant genetic interactions with other genes on chromosome 10. The bio-activation of clopidogrel is mediated by the CYP2C19 metabolic pathway and, therefore, *CYP2C19* genotypes are predictive of clopidogrel resistance [47]. Particularly, the CYP2C19*2 variant (rs4244285) has been associated with clopidogrel resistance in previous studies on Europeans [48,49]. However, rs4244285 was not among the top 10 signals in our study with Caribbean Hispanics. The rs7916697 SNP, an *ATHO7* intronic variant encoding a transcription factor for the regulation of the retinal ganglion cells and optic nerve, was among the 10 most significant variants selected instead. Notably, *ATHO7* is highly expressed in the liver (i.e., a GTEx value of 10, similar to that in brain tissues (GTEx = 12); https://www.genecards.org/cgi-bin/carddisp.pl?gene=ATOH7 (accessed on 10 September 2023)). Because this GTEx value is one of the highest across all tissues, this gene may be involved in the regulation of other gene expressions in the liver. However, the possible functional importance of other genes co-localized with the SNPs detected on chromosome 10 is unclear. We also selected the intronic variants rs4646743, rs4745950, and rs1900005, located within the region encoding an lncRNA called LINC02640, while the rs1900003 and rs1900002 variants located ~2 kb upstream of the LINC02640 were also considered. In addition, the intronic rs3796692 variant that is related to the ncRNA called LOC105377582 was included. Finally, the single-nucleotide variant rs4021557, located ~7 kb downstream of the *MAP2K3* gene involved in the regulation of the MAPK kinase pathway and MAPK kinase-mediated signaling cascades, was considered.

Reports of numerous SNPs associated with CVDs can be found in the available literature [38,50,51]. We identified 50 of these genetic variants significantly associated with the severity of CVDs (e.g., ACS, CAD, and/or PAD). These variants (Table 3) were associated with inflammatory pathways, cholesterol transport, and regulation and signaling cascades in previous GWASs [38,50,51]. Although most of these CVD-linked variants occur within intronic regions, they can either enhance or reduce gene expressions [52]. The eQTLs are genetic loci that alter gene expression and trait variation quantitatively [53]. These eQTL SNPs can change a quantitative trait through different mechanisms, such as epigenetic modifications, transcription, pre-mRNA processing, and post-transcriptional processing (mRNA stability and translation rate) [53].

BRG1 is part of the SWI/SNF complex, with ATPase and helicase activity, that participates in chromatin remodeling. Although the underlying pathway related to BRG1 is not yet fully elucidated, a recent human in vitro study demonstrated that BRG1 is highly expressed in hypertrophic cardiomyopathy and can alter myosin expression [54]. The rs9349379 SNP in the *PHACTR1* gene is an eQTL that has been found to be associated with CAD risk. PHACTR1 protein regulates the remodeling of the heart after myocardial infarction (MI), increasing its expression and the total protein levels after the event [55]. MI is commonly caused by coronary artery occlusion due to a thrombus; therefore, eQTL variants associated with thrombus formation (e.g., rs10455872, rs118039278, and rs140570886 in Table 3 and Table 4, and Table S4) are expected to be identified as signals of GWAS significance. These three SNPs have been correlated to one another for affecting the trait expression for lipoprotein A, a low-density lipoprotein (LDL) with apolipoprotein A, and have been associated with CAD and PAD [21,36,37,56,57]. The relevance of rs55791371 (Table 3 and Table 4, and Table S9) as an eQTL for the *SLC44A2* gene encoding the choline transporter was demonstrated in studies in vitro. Authors have suggested that ATP will be available for platelet aggregation by regulating the choline transport into the mitochondria through SLC44A2, thus resulting in a higher risk for thrombus formation [58].

Non-Random Enrichments: In this study, we found that relevant genetic risk loci associated with the severity of CVDs in Europeans are not enriched with SNPs linked to clopidogrel resistance among Caribbean Hispanic patients. Therefore, we did not identify a possible genetic marker with a dual role of predicting the severity of the CVDs and the response to clopidogrel in this target population. A possible explanation could be the limited number of SNPs to consider from the reference parental populations, narrowing the pool of possible variants to be studied. Databases containing genetic information from worldwide populations need to expand their variant catalog diversity, especially to account for relevant SNPs within the Native American populations.

Due to the emergence of novel bioinformatic tools, the genetically inferred ancestral composition of Caribbean Hispanics can now be better understood. To determine whether the enrichment was different from that observed in Caribbean Hispanics, the resulting ancestral enrichments at each of the 60 random SNPs were compared to those within the variant loci previously found to be associated with CVDs in Europeans. We found that the ancestral composition in the vicinity of the ten CVD-linked variants tested was similar to that around the 60 random variants. We speculate that certain locus-specific ancestry enrichments (e.g., African) at these risk loci can explain, to some extent, a possible breakup of the overlapping between SNPs related to clopidogrel resistance and the severity of CVDs or lack thereof in Caribbean Hispanics. The occurrence of novel haplotype blocks as a result of altered linkage disequilibrium (LD) patterns and the substantial admixture in Caribbean Hispanics might interfere with the expected associations between both types of SNPs.

Major Contribution: Our study is unique in that we carried out a targeted approach using bioinformatic tools and newly developed scripts to identify ancestry enrichments and non-random associations in a quite heterogeneous and admixed population of Caribbean Hispanic patients. Significant enrichments of African and Native American ancestry, but not of clopidogrel-associated genomic markers, were found within these 10 CVD-specific risk loci. We firmly believe that the ancestry enrichments observed at these 10 disease-specific relevant loci linked to the severity of CVDs will help predict the prevalence of significantly different inter-ethnic phenotypes even for highly admixed populations like Caribbean Hispanics. Consequently, such information could be of help for future admixture mapping studies in this population in order to refine the discovery of novel predictive signals by stratifying individuals regarding their ancestral enrichment at the locus of interest. That is, genetic loci showing high enrichment of ancestry related to the ethnic group with the highest prevalence of the surveyed phenotype will be targeted for more in-depth scrutiny, as long as the phenotype shows differential risk by ancestry.

Recently admixed populations, like Caribbean Hispanics, have a genome that is reminiscent of a mosaic, with sections inherited from Africa and others inherited from Europe or native populations. Consequently, genetically inferred global and local ancestry is an important consideration in the drug response specifically in admixed populations at the gene level. The Hispanic population is highly admixed, but their ancestral proportions differ across different ethno-geographic locations due to historical context and other determinants. This admixture highlights challenges for the current CPIC guideline for clopidogrel selection, which does not discriminate between patients with different ethnic-geographic backgrounds. For example, should any amount of enrichment with European ancestry or certain CVD-linked risk loci require an individual to obtain CYP2C19 genotyping prior to benefiting from PGx-guided antiplatelet therapy? Therefore, our results could also help to delineate optimal strategies for the individualization of antiplatelet therapies based on the ancestral origin and non-random CVD-related enrichment of patients at risk.

Study limitations include a relatively modest sample size in the surveyed cohort when compared to the usual larger number of participants in similar studies within other continental populations (i.e., 2750 Europeans in the International Clopidogrel Pharmacogenomics Consortium (ICPC) study) [59]. However, our patients represent a more diverse group than that of the ICPC study. Ancestral proportions, both local and global, are inherently biased due to the European-centered nature of the reference genomes used for their inferences [60]. Although of minimal impact, collection biases due to the potential for patient noncompliance and the exclusion of stroke patients are additional limitations of this analysis. Finally, our work did not include data regarding the use of alternative antiplatelet drugs (e.g., prasugrel, ticagrelor), which may be preferred in many clinical scenarios.

## 5. Conclusions

In conclusion, even though we did not find a non-random enrichment of CVD-associated variants within sites nearby those associated with clopidogrel resistance, there are still possible novel ethnic-specific variants to be discovered and added to reference panels for future inquiries. Our data bring insightful information about the locus-specific ancestry in Caribbean Hispanics, with Native American ancestry being the most enriched within risk loci for variants linked to CVDs. The non-random enrichment of disease susceptibility loci with SNPs related to drug responsiveness needs further attention to improve the experimental designs of studies seeking the adoption of the Precision Medicine paradigm.

The use of valid bioinformatic tools to identify novel or overlooked eQTLs linked to critical biological pathways in this admixed population may further unravel relevant biomarkers of CVD severity, as well as of clopidogrel resistance. We strongly believe that the integration of information about ancestry and genetic risk variants can expedite the identification of underlying disease-related mechanisms in Caribbean Hispanics. Because most of the published studies identifying SNPs linked to CVDs (i.e., ACS, CAD, and PAD) were conducted on mainly Europeans, the assessment of a potential non-random enrichment of SNPs associated with clopidogrel resistance within risk loci linked to the CVD severity in a cohort of Caribbean Hispanics, and the comparison of LAIs at 10 CVD risk loci and 60 additional randomly selected SNPs, will also contribute to further addressing the existing health disparity among Caribbean Hispanics.

## Figures and Tables

**Table 1:** Inclusion and exclusion criteria.

Inclusion Criteria	Exclusion Criteria
Caribbean Hispanics residing in Puerto Rico Both genders (males/females) Age ≥ 21 Receiving clopidogrel (75 mg/day) for these therapeutic indications: ACS, CAD, and PAD ^#^ No clinically active hepatic abnormality The ability to understand the requirements of the study The ability to comply with the study procedures and protocols A female patient is eligible to enter the study if she is of child-bearing potential and not pregnant or nursing, or not of child-bearing potential	Non-Hispanic patients Currently enrolled in another active research protocol Other therapeutic indications ^#^ BUN > 30 and creatinine > 2.0 mg/dL Hematocrit (Hct) ≤ 25% Nasogastric or enteral feedings Acute illness (e.g., sepsis, infection, anemia) HIV/AIDS, hepatitis B patients Alcoholism and drug abuse Patients with any cognitive and mental health impairment Sickle cell patients Active malignancy Patients taking another antiplatelet

# Because less than 5% of the participants in the study cohort received clopidogrel for indications other than ACS, CAD, or PAD (i.e., a stroke or transient ischemic attack (TIA)), we decided not to include these patients in further analyses. Furthermore, the role of genomics and the impact of specific genetic markers on the clopidogrel response in stroke patients is less well understood [21].

**Table 2:** Characteristics of participants in the study cohort.

Variable	Mean	SD	SEM	Min.	Max.	Median
Age (years)	68.01	10.95	0.51	27.00	94.00	69.00
BMI (kg/m^2^)	28.40	5.71	0.27	11.48	52.67	27.67
**Variable**			***n***		**%**	
Gender (males)			282		55.29	
Diabetes mellitus			280		54.79	
Hypertension			428		83.92	
Dyslipidemias			367		71.96	
Smoking			69		13.53	
MACE ^¶^			42		13.77	
MIs ^¶^			19		6.23	
Stent thrombosis ^¶^			15		4.92	
Deaths ^¶^			4		1.31	
Bleeding events *			83		16.24	
Aspirin use			323		63.32	
Statin use			404		79.21	
CCBs			137		26.86	
PPIs			102		19.96	
LVEF ≤ 30%			42		8.23	
ACS and stable CAD			387		75.88	
Coronary artery stenting ^#^			191		37.45	
PAD			123		24.12	

Note: Due to rounding errors, percentages may not equal 100%. ^¶^ MACE reports available only in a subset of 305 patients. * Bleeding events are a combination of major and minor events. ^#^ All patients with ACS and some with CAD underwent PCI (i.e., percutaneous coronary intervention) or coronary artery stenting. MIs: myocardial infarctions (i.e., STEMI and NSTEMI). CCBs: calcium channel blockers. PPIs: proton pump inhibitors. BMI: body mass index. PAD: peripheral artery disease.

**Table 3:** Identified SNPs associated with ACS, CAD, and PAD in previous literature and SNPs related to clopidogrel resistance in Caribbean Hispanics.

Chr	POS	SNP	Relation	Reference
19	45412079	rs7412	ACS	[23]
6	46677098	rs76863441	ACS	[23]
1	109817590	rs12740374	ACS	[23]
20	23616469	rs35610040	ACS	[24]
7	99421085	rs62471956	ACS	[25]
6	161010118	rs10455872	ACS	[23]
15	44408401	rs2733201	ACS	[23]
15	44293137	rs11638352	ACS	[23]
6	161111700	rs186696265	ACS	[23]
7	99286639	rs188845491	ACS	[25]
7	98932759	rs147642358	ACS	[25]
7	100103523	rs140607780	ACS	[25]
6	160751531	rs9295128	ACS	[23]
7	99543627	rs140104968	ACS	[25]
14	84804488	rs117714106	ACS	[23]
7	99841354	rs117038461	ACS	[25]
15	44564692	rs144972973	ACS	[23]
1	172995643	rs201052613	ACS	[23]
12	125307053	rs11057830	ACS	[23]
9	107396924	rs189889864	ACS	[23]
6	161013013	rs140570886	CAD	[26,27]
6	12903957	rs9349379	CAD	[26,27,28,29,30,31,32,33,34,35]
1	109821511	rs602633	CAD	[27,36,37,38]
19	11202306	rs6511720	CAD	[23,27,34,37]
19	11188153	rs55791371	CAD	[27,31,36]
15	79141784	rs7173743	CAD	[27,31,36]
21	35593827	rs28451064	CAD	[26,27,30,34,35,37,39]
2	203873743	rs6728861	CAD	[27]
2	203968973	rs72934535	CAD	[27,37,39]
2	203893999	rs115654617	CAD	[27]
6	160911596	rs147555597	CAD	[27]
6	134209837	rs2327429	CAD	[27,31,40]
6	161018174	rs7770628	CAD	[41]
12	111884608	rs3184504	CAD	[27,35,36,37,42]
11	103660567	rs974819	CAD	[27,28]
6	134159622	rs1966248	CAD	[27]
1	222829550	rs35158675	CAD	[27,31]
1	56966350	rs17114046	CAD	[27,28,39]
1	222837939	rs17465982	CAD	[27]
12	111932800	rs7137828	CAD	[27,37]
6	160985526	rs118039278	PAD	[26,43]
9	22103183	rs1537372	PAD	[26,43]
15	78915864	rs10851907	PAD	[26,43]
1	169519049	rs6025	PAD	[26,43]
7	19049388	rs2107595	PAD	[26,43,44]
1	109817192	rs7528419	PAD	[26,43]
12	112871372	rs11066301	PAD	[26,43]
7	22786532	rs4722172	PAD	[26,43]
10	114758349	rs7903146	PAD	[26,43]
9	136149229	rs505922	PAD	[26,43]
10	69996292	rs4746743	Clopidogrel	This study
10	69998055	rs1900005	Clopidogrel	This study
10	69999026	rs12098677	Clopidogrel	This study
21	39485558	rs9980291	Clopidogrel	This study
10	70004551	rs1900003	Clopidogrel	This study
10	70004552	rs1900002	Clopidogrel	This study
10	69996455	rs4745950	Clopidogrel	This study
4	185205210	rs3796692	Clopidogrel	This study
17	21225519	rs4021557	Clopidogrel	This study
10	69991853	rs7916697	Clopidogrel	This study

Chr: chromosome; POS: position in GRCh37; SNP: single-nucleotide polymorphism. Position is based on human assembly GRCh37/h19 genome reference build by the Genome Reference Consortium.

**Table 4:** Ancestry search for SNPs associated with cardiovascular conditions in previous literature reports.

rs Number	Chr	POS	cM	IBS	YRI	NA
rs7528419	1	109817192	138.712247	0.064	0.09053	0.84549
rs17465982	1	222837939	246.478996	0.0074	0.67445	0.3182
rs9349379	6	12903957	28.8898785	0.0172	0.41137	0.57144
rs10455872	6	161010118	177.557204	0.5659	0.41854	0.01556
rs4722172	7	22786532	40.0928349	0.0329	0.39048	0.57665
rs11066301	12	112871372	130.305862	0.1981	0.00386	0.79809
rs11057830	12	125307053	149.857655	0.1827	0.16692	0.65043
rs10851907	15	78915864	102.193041	0.0029	0.45321	0.54385
rs55791371	19	11188153	31.8736735	0.0027	0.08713	0.91016
rs28451064	21	35593827	36.2865085	0.0789	0.51911	0.40204

**Table 5:** Ancestry enrichment for 60 random SNPs generated by computational tools.

rs Number	Chr	POS	cM	IBS	YRI	NA
rs62394277	5	169924749	185.487416	0.23	0.39	0.37
rs12033818	1	106368717	134.610925	0.05	0.01	0.94
rs10960270	9	11818115	25.717268	0.19	0.24	0.56
rs2471660	12	75534099	89.727097	0.00	0.79	0.21
rs12627722	21	17918366	7.52177123	0.15	0.70	0.15
rs11653073	17	55687365	83.7730559	0.00	0.41	0.59
rs17025629	3	88354232	109.781656	0.67	0.22	0.11
rs7578180	2	158772058	176.901137	0.10	0.56	0.34
rs75910375	17	11281457	29.8450381	0.01	0.49	0.50
rs74936620	1	55832969	81.3389065	0.12	0.24	0.64
rs77240763	5	145752501	153.573126	0.31	0.31	0.38
rs75970478	3	138843097	150.937494	0.11	0.62	0.26
rs12956301	18	71834423	105.245876	0.00	0.80	0.20
rs10172437	2	174701949	193.753831	0.01	0.60	0.39
rs56919396	14	95350520	95.3439121	0.36	0.16	0.48
rs2567778	13	103742001	103.442192	0.36	0.52	0.12
rs17128958	14	93708224	91.6536648	0.42	0.14	0.44
rs576400715	2	89571430	120.624	0.05	0.30	0.65
rs17089367	13	73011013	70.4386922	0.30	0.60	0.10
rs10948131	6	44291641	69.8263172	0.00	0.57	0.43
rs114354246	3	137295824	149.759253	0.15	0.61	0.24
rs7128963	11	33599446	50.7219531	0.06	0.29	0.66
rs6691697	1	42534375	70.0178338	0.07	0.32	0.61
rs12060034	1	198224252	215.353224	0.28	0.21	0.51
rs12205759	6	122752471	127.395537	0.04	0.43	0.54
rs1611636	6	29836703	50.3216201	0.04	0.32	0.64
rs10512946	3	134916937	148.665958	0.15	0.61	0.23
rs72866243	18	2094235	5.55281054	0.45	0.44	0.11
rs114208611	11	11100541	21.4527621	0.00	0.40	0.60
rs13136539	4	174513213	183.196957	0.47	0.26	0.27
rs77282632	3	121384210	133.9296	0.43	0.30	0.27
rs730489	6	151399891	162.916228	0.29	0.48	0.23
rs528255	8	12878637	33.8526902	0.10	0.51	0.39
rs72834798	17	38217299	64.1399895	0.01	0.56	0.43
rs16883890	5	9950908	22.6794291	0.12	0.08	0.80
rs11996214	8	135211378	159.947948	0.01	0.60	0.39
rs4782152	16	9326011	22.425679	0.01	0.13	0.86
rs13432204	2	107262432	128.94	0.05	0.20	0.74
rs16974292	16	84652644	114.995507	0.03	0.30	0.67
rs116953645	19	46613411	72.3867019	0.01	0.31	0.67
rs2422672	20	1990286	7.8190193	0.10	0.70	0.20
rs17188743	6	30385111	50.6059471	0.05	0.32	0.63
rs973295	14	94372768	92.5873731	0.39	0.15	0.46
rs1889877	6	69729678	84.7215414	0.01	0.48	0.51
rs10114476	9	96631459	113.576597	0.15	0.71	0.14
rs113629052	9	95358140	112.692987	0.14	0.71	0.15
rs62568088	9	6277534	15.8022578	0.12	0.32	0.56
rs4631137	5	52420445	63.8860104	0.04	0.56	0.40
rs2165183	2	4830190	9.36070063	0.11	0.67	0.21
rs675465	4	20543817	36.4528078	0.33	0.27	0.40
rs76887708	5	98125372	109.316657	0.00	0.76	0.24
rs8043824	16	79847142	101.522556	0.01	0.31	0.68
rs17123256	14	87745773	81.7304956	0.45	0.36	0.19
rs115079994	22	45016212	55.6235323	0.01	0.07	0.92
rs113899132	5	156284101	166.222937	0.17	0.31	0.52
rs73142595	20	54320992	85.8174786	0.21	0.22	0.57
rs7700639	5	6606807	16.6711274	0.20	0.01	0.79
rs7714907	5	142145386	149.584982	0.28	0.37	0.35
rs114959453	6	24423918	47.4334264	0.03	0.35	0.62
rs9812688	3	86878962	109.260905	0.67	0.22	0.11

Chr: chromosome; POS: position in GRCh37; cM: centimorgan. Ancestry data are presented as the average from IBS (Iberian population in Spain), YRI (Yoruba in Ibadan, Nigeria), and NA (American population) of 510 Caribbean Hispanic patients. Position is based on the human assembly GrCh37/h19 genome reference build by the Genome Reference Consortium.

## Data Availability

The data used as the reference populations are Surui and Karitiana in Brazil, Piapoco and Colombian in Colombia, and Maya and Pima in Mexico, found at https://www.internationalgenome.org/data-portal/data-collection/hgdp (accessed on 7 October 2021). IBS and YRI populations are from the NHGRI Sample Repository for Human Genetic Research of the Coriell Institute found at https://www.internationalgenome.org/data-portal/population (accessed on 7 October 2021). The datasets from the Caribbean Hispanics analyzed during the current study are available from the corresponding author upon request.

## Supplementary Materials

The following supporting information can be downloaded at https://www.mdpi.com/article/10.3390/genes14091813/s1, Figure S1: Flow diagram of the study design. Additional file 1 named gtex_eqtl.xlsx is in .xls format and includes the following tables: Table S1: eQTLs from SNPs on chromosome 1 associated with cardiovascular conditions; Table S2: eQTLs from SNPs on chromosome 2 associated with cardiovascular conditions; Table S3: eQTLs from SNPs on chromosome 6 associated with cardiovascular conditions; Table S4: eQTLs from SNPs on chromosome 7 associated with cardiovascular conditions; Table S5: eQTLs from SNPs on chromosome 10 associated with cardiovascular conditions and resistance to clopidogrel in Caribbean Hispanics; Table S6: eQTLs from SNPs on chromosome 11 associated with cardiovascular conditions; Table S7: eQTLs from SNPs on chromosome 12 associated with cardiovascular conditions; Table S8: eQTLs from SNPs on chromosome 15 associated with cardiovascular conditions; Table S9: eQTLs from SNPs on chromosome 19 associated with cardiovascular conditions; Table S10: eQTLs from SNPs on chromosome 20 associated with cardiovascular conditions; Table S11: eQTLs from SNPs on chromosome 21 associated with cardiovascular conditions.

## Abbreviations

ACS	acute coronary syndrome
CAD	coronary artery disease
CV	cardiovascular
CHR	chromosome
cM	centimorgan
DAPT	dual antiplatelet therapy
eQTL	expression quantitative trait loci
GWAS	genome-wide association study
Hct	hematocrit
HGDP	Human Genome Diversity Project
HTPR	high on-treatment platelet reactivity
IBS	Iberian
LA	local ancestry
LAI	local-ancestry inference
LDL	low-density lipoprotein
MEGA	Multi-Ethnic AMR/AFR Genotyping Array
MI	myocardial infarction
NA	Native American
PAD	peripheral artery disease
PHACTR1	phosphatase and actin regulator 1
POS	position
SNP	single-nucleotide polymorphism
TIA	transient ischemic attack
YRI	Yoruba

## References

[1] Sirugo G., Williams S.M., Tishkoff S.A. (2019). The Missing Diversity in Human Genetic Studies. Cell.

[2] Evangelou E., Program T.M.V., Warren H.R., Mosen-Ansorena D., Mifsud B., Pazoki R., Gao H., Ntritsos G., Dimou N., Cabrera C.P. (2018). Genetic analysis of over 1 million people identifies 535 new loci associated with blood pressure traits. Nat. Genet..

[3] Johnson A.D., Yanek L.R., Chen M.-H., Faraday N., Larson M.G., Tofler G., Lin S.J., Kraja A.T., Province M.A., Yang Q. (2010). Genome-wide meta-analyses identifies seven loci associated with platelet aggregation in response to agonists. Nat. Genet..

[4] Hernandez-Suarez D.F., Botton M.R., Scott S., Tomey M., Garcia M.J., Wiley J., Villablanca P., Melin K., Lopez-Candales A., Renta J.Y. (2018). Pharmacogenetic association study on clopidogrel response in Puerto Rican Hispanics with cardiovascular disease: A novel characterization of a Caribbean population. Pharmacogenomics Pers. Med..

[5] Hernandez-Suarez D.F., Scott S.A., Tomey M.I., Melin K., Lopez-Candales A., Buckley C.E., Duconge J. (2017). Clinical determinants of clopidogrel responsiveness in a heterogeneous cohort of Puerto Rican Hispanics. Ther. Adv. Cardiovasc. Dis..

[6] Tcheng J.E., Lim I.H., Srinivasan S., Jozic J., Gibson C.M., O’Shea J.C., Puma J.A., Simon D. (2009). Stent parameters predict major adverse clinical events and the response to platelet glycoprotein IIb/IIIa blockade: Findings of the ESPRIT trial. Circ. Cardiovasc. Interv..

[7] Janssen P.W.A., Bergmeijer T.O., Vos G.-J.A., Kelder J.C., Qaderdan K., Godschalk T.C., Breet N.J., Deneer V.H.M., Hackeng C.M., Berg J.M.T. (2019). Tailored P2Y12 inhibitor treatment in patients undergoing non-urgent PCI—The POPular Risk Score study. Eur. J. Clin. Pharmacol..

[8] Via M., Gignoux C.R., Roth L.A., Fejerman L., Galanter J., Choudhry S., Toro-Labrador G., Viera-Vera J., Oleksyk T.K., Beckman K. (2011). History Shaped the Geographic Distribution of GenomicAdmixture on the Island of Puerto Rico. PLoS ONE.

[9] Hernández C.L., Pita G., Cavadas B., López S., Sánchez-Martínez L.J., Dugoujon J.-M., Novelletto A., Cuesta P., Pereira L., Calderón R. (2020). Human Genomic Diversity Where the Mediterranean Joins the Atlantic. Mol. Biol. Evol..

[10] Shriner D., Adeyemo A., Ramos E., Chen G., Rotimi C.N. (2011). Mapping of disease-associated variants in admixed populations. Genome Biol..

[11] Torgerson D.G., Gignoux C.R., Galanter J.M., Drake K.A., Roth L.A., Eng C., Huntsman S., Torres R., Avila P.C., Chapela R. (2012). Case-control admixture mapping in Latino populations enriches for known asthma-associated genes. J. Allergy Clin. Immunol..

[12] Baran Y., Pasaniuc B., Sankararaman S., Torgerson D.G., Gignoux C., Eng C., Rodriguez-Cintron W., Chapela R., Ford J.G., Avila P.C. (2012). Fast and accurate inference of local ancestry in Latino populations. Bioinformatics.

[13] Uren C., Hoal E.G., Möller M. (2020). Putting RFMix and ADMIXTURE to the test in a complex admixed population. BMC Genet..

[14] Guan Y. (2014). Detecting Structure of Haplotypes and Local Ancestry. Genetics.

[15] Salter-Townshend M., Myers S. (2019). Fine-scale inference of ancestry segments without prior knowledge of admixing groups. Genetics.

[16] Schubert R., Andaleon A., Wheeler H.E. (2020). Comparing local ancestry inference models in populations of two- and three-way admixture. PeerJ.

[17] Maples B.K., Gravel S., Kenny E.E., Bustamante C.D. (2013). RFMix: A Discriminative Modeling Approach for Rapid and Robust Local-Ancestry Inference. Am. J. Hum. Genet..

[18] Browning S.R., Grinde K., Plantinga A., Gogarten S.M., Stilp A.M., Kaplan R.C., Avilés-Santa M.L., Browning B.L., Laurie C.C. (2016). Local ancestry inference in a large US-based Hispanic/Latino study: Hispanic community health study/study of Latinos (HCHS/SOL). G3 Genes Genomes Genet..

[19] Sofer T., Baier L.J., Browning S.R., Thornton T.A., Talavera G.A., Wassertheil-Smoller S., Daviglus M.L., Hanson R., Kobes S., Cooper R.S. (2017). Admixture mapping in the Hispanic Community Health Study/Study of Latinos reveals regions of genetic associations with blood pressure traits. PLoS ONE.

[20] Duconge J., Santiago E., Hernandez-Suarez D.F., Moneró M., López-Reyes A., Rosario M., Renta J.Y., González P., Fernández-Morales L.I., Vélez-Figueroa L.A. (2021). Pharmacogenomic polygenic risk score for clopidogrel responsiveness among Caribbean Hispanics: A candidate gene approach. Clin. Transl. Sci..

[21] Zhuang Z., Yao M., Wong J.Y.Y., Liu Z., Huang T. (2021). Shared genetic etiology and causality between body fat percentage and cardiovascular diseases: A large-scale genome-wide cross-trait analysis. BMC Med..

[22] Buniello A., MacArthur J.A.L., Cerezo M., Harris L.W., Hayhurst J., Malangone C., McMahon A., Morales J., Mountjoy E., Sollis E. (2019). The NHGRI-EBI GWAS Catalog of published genome-wide association studies, targeted arrays and summary statistics 2019. Nucleic Acids Res..

[23] Yeo A., Li L., Warren L., Aponte J., Fraser D., King K., Johansson K., Barnes A., MacPhee C., Davies R. (2017). Pharmacogenetic meta-analysis of baseline risk factors, pharmacodynamic, efficacy and tolerability endpoints from two large global cardiovascular outcomes trials for darapladib. PLoS ONE.

[24] Åkerblom A., Eriksson N., Wallentin L., Siegbahn A., Barratt B.J., Becker R.C., Budaj A., Himmelmann A., Husted S., Storey R.F. (2014). Polymorphism of the cystatin C gene in patients with acute coronary syndromes: Results from the PLATelet inhibition and patient Outcomes study. Am. Heart J..

[25] Varenhorst C., Eriksson N., Johansson A.A.S.A., Barratt B.J., Hagström E., Åkerblom A., Syvänen A.-C., Becker R.C., James S.K., Katus H.A. (2015). Effect of genetic variations on ticagrelor plasma levels and clinical outcomes. Eur. Heart J..

[26] Klarin D., Zhu Q.M., Emdin C.A., Chaffin M., Horner S., McMillan B.J., Leed A., Weale M.E., Spencer C.C., Aguet F. (2017). Genetic analysis in UK Biobank links insulin resistance and transendothelial migration pathways to coronary artery disease. Nat. Genet..

[27] van der Harst P., Verweij N. (2018). Identification of 64 Novel Genetic Loci Provides an Expanded View on the Genetic Architecture of Coronary Artery Disease. Circ. Res..

[28] Mehta N.N. (2011). A Genome-Wide Association Study in Europeans and South Asians Identifies 5 New Loci for Coronary Artery Disease. Circ. Cardiovasc. Genet..

[29] Zhong W.-P., Wu H., Chen J.-Y., Li X.-X., Lin H.-M., Zhang B., Zhang Z.-W., Ma D.-L., Sun S., Li H.-P. (2017). Genomewide Association Study Identifies Novel Genetic Loci That Modify Antiplatelet Effects and Pharmacokinetics of Clopidogrel. Clin. Pharmacol. Ther..

[30] Saw J., Yang M.-L., Trinder M., Tcheandjieu C., Xu C., Starovoytov A., Birt I., Mathis M.R., Hunker K.L., Schmidt E.M. (2020). Chromosome 1q21.2 and additional loci influence risk of spontaneous coronary artery dissection and myocardial infarction. Nat. Commun..

[31] Koyama S., Ito K., Terao C., Akiyama M., Horikoshi M., Momozawa Y., Matsunaga H., Ieki H., Ozaki K., Onouchi Y. (2020). Population-specific and trans-ancestry genome-wide analyses identify distinct and shared genetic risk loci for coronary artery disease. Nat. Genet..

[32] Hager J., Kamatani Y., Cazier J.-B., Youhanna S., Ghassibe-Sabbagh M., Platt D.E., Abchee A.B., Romanos J., Khazen G., Othman R. (2012). Genome-Wide Association Study in a Lebanese Cohort Confirms PHACTR1 as a Major Determinant of Coronary Artery Stenosis. PLoS ONE.

[33] Lu X., Wang L., Chen S., He L., Yang X., Shi Y., Cheng J., Zhang L., Gu C.C., The Coronary ARtery DIsease Genome-Wide Replication And Meta-Analysis (CARDIoGRAM) Consortium (2012). Genome-wide association study in Han Chinese identifies four new susceptibility loci for coronary artery disease. Nat. Genet..

[34] Nelson C.P., Goel A., Butterworth A.S., Kanoni S., Webb T.R., Marouli E., Zeng L., Ntalla I., Lai F.Y., Hopewell J.C. (2017). Association analyses based on false discovery rate implicate new loci for coronary artery disease. Nat. Genet..

[35] Nikpay M., Goel A., Won H.H., Hall L.M. (2015). A comprehensive 1000 Genomes-based genome-wide association meta-analysis of coronary artery disease. Nat. Genet..

[36] Dichgans M., Rainer M., König I.R. (2013). Shared Genetic Susceptibility to Ischemic Stroke and Coronary Artery Disease: A Ge-nome-Wide Analysis of Common Variants. Stroke.

[37] Temprano-Sagrera G., Sitlani C.M., Bone W.P., Martin-Bornez M., Voight B.F., Morrison A.C., Damrauer S.M., de Vries P.S., Smith N.L., Sabater-Lleal M. (2022). Multi-phenotype analyses of hemostatic traits with cardiovascular events reveal novel genetic associations. J. Thromb. Haemost..

[38] Vujkovic M., Keaton J.M., Lynch J.A., Miller D.R., Zhou J., Tcheandjieu C., Huffman J.E., Assimes T.L., Lorenz K., Zhu X. (2020). Discovery of 318 new risk loci for type 2 diabetes and related vascular outcomes among 1.4 million participants in a multi-ancestry meta-analysis. Nat. Genet..

[39] Zhou W., Nielsen J.B., Fritsche L.G., Dey R., Gabrielsen M.E., Wolford B.N., LeFaive J., VandeHaar P., Gagliano S.A., Gifford A. (2018). Efficiently controlling for case-control imbalance and sample relatedness in large-scale genetic association studies. Nat. Genet..

[40] Matsunaga H., Ito K., Akiyama M., Takahashi A., Koyama S., Nomura S., Ieki H., Ozaki K., Onouchi Y., Sakaue S. (2020). Transethnic Meta-Analysis of Genome-Wide Asso-ciation Studies Identifies Three New Loci and Characterizes Population-Specific Differences for Coronary Artery Disease. Circ. Genom. Precis. Med..

[41] Liu Y., Ma H., Zhu Q., Zhang B., Yan H., Li H., Meng J., Lai W., Li L., Yu D. (2019). A genome-wide association study on lipoprotein (a) levels and coronary artery disease severity in a Chinese population. J. Lipid Res..

[42] Schunkert H., König I.R., Kathiresan S., Reilly M.P., Assimes T.L., Holm H., Preuss M., Stewart A.F., Barbalic M., Gieger C. (2011). Large-scale association analysis identifies 13 new susceptibility loci for coronary artery disease. Nat. Genet..

[43] Van Zuydam N.R., Stiby A., Abdalla M., Austin E., Dahlström E.H., McLachlan S., Vlachopoulou E., Ahlqvist E., Di Liao C., Sandholm N. (2021). Genome-Wide Association Study of Peripheral Artery Disease. Circ. Genom. Precis. Med..

[44] Ishigaki K., Akiyama M., Kanai M., Takahashi A., Kawakami E., Sugishita H., Sakaue S., Matoba N., Low S.-K., Okada Y. (2020). Large-scale genome-wide association study in a Japanese population identifies novel susceptibility loci across different diseases. Nat. Genet..

[45] Fall T., Gustafsson S., Orho-Melander M., Ingelsson E. (2018). Genome-wide association study of coronary artery disease among individuals with diabetes: The UK Biobank. Diabetologia.

[46] Ward-Caviness C.K., Neas L.M., Blach C., Haynes C.S., LaRocque-Abramson K., Grass E., Dowdy E., Devlin R.B., Diaz-Sanchez D., Cascio W.E. (2016). Genetic variants in the bone mor-phogenic protein gene family modify the association between residential exposure to traffic and peripheral arterial disease. PLoS ONE.

[47] Lee C.R., Luzum J.A., Sangkuhl K., Gammal R.S., Sabatine M.S., Stein C.M., Kisor D.F., Limdi N.A., Lee Y.M., Scott S.A. (2022). Clinical Pharmacogenetics Implementation Consortium Guideline for *CYP2C19* Genotype and Clopidogrel Therapy: 2022 Update. Clin. Pharmacol. Ther..

[48] Kazui M., Nishiya Y., Ishizuka T., Hagihara K., Farid N.A., Okazaki O., Ikeda T., Kurihara A. (2010). Identification of the Human Cytochrome P450 Enzymes Involved in the Two Oxidative Steps in the Bioactivation of Clopidogrel to Its Pharmacologically Active Metabolite. Drug Metab. Dispos..

[49] Hulot J.S., Bura A., Villard E., Azizi M., Remones V., Goyenvalle C., Aiach M., Lechat P., Gaussem P. (2006). Cytochrome P450 2C19 loss-of-function polymorphism is a major determinant of clopidogrel responsiveness in healthy subjects. Blood.

[50] Yamada Y., Yasukochi Y., Kato K., Oguri M., Horibe H., Fujimaki T., Takeuchi I., Sakuma J. (2018). Identification of 26 novel loci that confer susceptibility to early-onset coronary artery disease in a Japanese population. Biomed. Rep..

[51] Liu X., Xu H., Xu H., Geng Q., Mak W.H., Ling F., Su Z., Yang F., Zhang T., Chen J. (2021). New genetic variants associated with major adverse cardiovascular events in patients with acute coronary syndromes and treated with clopidogrel and aspirin. Pharmacogenomics J..

[52] Shaul O. (2017). How introns enhance gene expression. Int. J. Biochem. Cell Biol..

[53] Pai A.A., Pritchard J.K., Gilad Y. (2015). The Genetic and Mechanistic Basis for Variation in Gene Regulation. PLoS Genet..

[54] Scherba J.C., Halushka M.K., Andersen N.D., Maleszewski J.J., Landstrom A.P., Bursac N., Glass C. (2022). BRG1 is a biomarker of hypertrophic cardiomyopathy in human heart specimens. Sci. Rep..

[55] Kelloniemi A., Szabo Z., Serpi R., Näpänkangas J., Ohukainen P., Tenhunen O., Kaikkonen L., Koivisto E., Bagyura Z., Kerkelä R. (2015). The Early-Onset Myocardial Infarction Associated PHACTR1 Gene Regulates Skeletal and Cardiac Alpha-Actin Gene Expression. PLoS ONE.

[56] Klarin D., Program V.M.V., Lynch J., Aragam K., Chaffin M., Assimes T.L., Huang J., Lee K.M., Shao Q., Huffman J.E. (2019). Genome-wide association study of peripheral artery disease in the Million Veteran Program. Nat. Med..

[57] Shah S., Henry A., Roselli C., Lin H., Sveinbjörnsson G., Fatemifar G., Hedman Å.K., Wilk J.B., Morley M.P., Chaffin M.D. (2020). Genome-wide association, and Mendelian randomisation analysis provide insights into the pathogenesis of heart failure. Nat. Commun..

[58] Bennett J.A., Mastrangelo M.A., Ture S.K., Smith C.O., Loelius S.G., Berg R.A., Shi X., Burke R.M., Spinelli S.L., Cameron S.J. (2020). The choline transporter Slc44a2 controls platelet activation and thrombosis by regulating mitochondrial function. Nat. Commun..

[59] Verma S.S., Bergmeijer T.O., Gong L., Reny J., Lewis J.P., Mitchell B.D., Alexopoulos D., Aradi D., Altman R.B., ICPC Investigators (2020). Genomewide Association Study of Platelet Reactivity and Cardiovascular Response in Patients Treated with Clopidogrel: A Study by the International Clopidogrel Pharmacogenomics Consortium. Clin. Pharmacol. Ther..

[60] Levy-Sakin M., Pastor S., Mostovoy Y., Li L., Leung A.K.Y., McCaffrey J., Young E., Lam E.T., Hastie A.R., Wong K.H.Y. (2019). Genome maps across 26 human populations reveal population-specific patterns of structural variation. Nat. Commun..

