# Supplementary figures and images for: Non-Random Enrichment of Single-Nucleotide Polymorphisms Associated with Clopidogrel Resistance within Risk Loci Linked to the Severity of Underlying Cardiovascular Diseases: The Role of Admixture
Source: Genes (Basel). 2023 Sep 17;14(9):1813. doi: 10.3390/genes14091813 (PMC10531115; doi:10.3390/genes14091813)

**Figure S1. Flow diagram of the study design.**

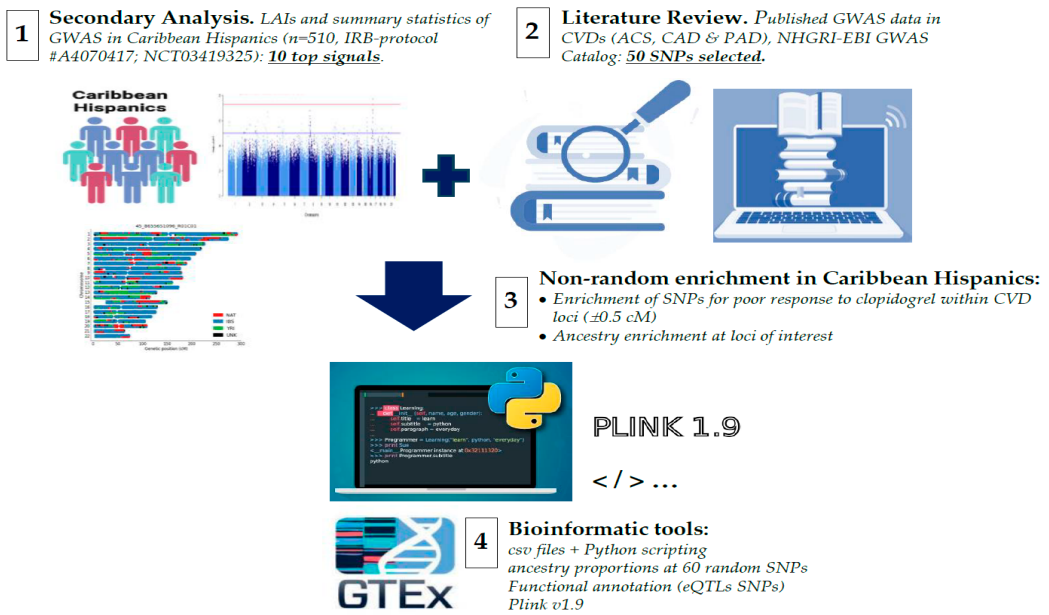

Supplement: Supplementary file 1 [file genes-14-01813-s001.zip › genes-2611583-Figure S1.pdf]
